# An Evolutionary Classification of Genomic Function

**DOI:** 10.1093/gbe/evv021

**Published:** 2015-01-28

**Authors:** Dan Graur, Yichen Zheng, Ricardo B.R. Azevedo

**Affiliations:** Department of Biology and Biochemistry, University of Houston

**Keywords:** Functional DNA, literal DNA, indifferent DNA, rubbish DNA, junk DNA, garbage DNA, pseudogene, Lazarus DNA, zombie DNA, Jekyll-to-Hyde DNA

## Abstract

The pronouncements of the ENCODE Project Consortium regarding “junk DNA” exposed the need for an evolutionary classification of genomic elements according to their selected-effect function. In the classification scheme presented here, we divide the genome into “functional DNA,” that is, DNA sequences that have a selected-effect function, and “rubbish DNA,” that is, sequences that do not. Functional DNA is further subdivided into “literal DNA” and “indifferent DNA.” In literal DNA, the order of nucleotides is under selection; in indifferent DNA, only the presence or absence of the sequence is under selection. Rubbish DNA is further subdivided into “junk DNA” and “garbage DNA.” Junk DNA neither contributes to nor detracts from the fitness of the organism and, hence, evolves under selective neutrality. Garbage DNA, on the other hand, decreases the fitness of its carriers. Garbage DNA exists in the genome only because natural selection is neither omnipotent nor instantaneous. Each of these four functional categories can be 1) transcribed and translated, 2) transcribed but not translated, or 3) not transcribed. The affiliation of a DNA segment to a particular functional category may change during evolution: Functional DNA may become junk DNA, junk DNA may become garbage DNA, rubbish DNA may become functional DNA, and so on; however, determining the functionality or nonfunctionality of a genomic sequence must be based on its present status rather than on its potential to change (or not to change) in the future. Changes in functional affiliation are divided into pseudogenes, Lazarus DNA, zombie DNA, and Jekyll-to-Hyde DNA.

## Introduction

Genomic sequences are frequently categorized according to biochemical activity, regardless of whether or not such activity is biologically meaningful. Two erroneous equivalencies are particularly common. The first equivalency, usually espoused in the medical literature, erroneously equates “noncoding DNA”—that is, all regions in the genome that do not encode proteins—with “junk DNA”—that is, all regions in the genome that are neither functional nor deleterious (e.g., Krams and Bromberg 2013; Mehta et al. 2013). The second, more pernicious equivalency transmutes every biochemical activity into a function (e.g., ENCODE Project Consortium 2012; Sundaram et al. 2014; Kellis et al. 2014). Distinguishing between what a genomic element does (its causal-role activity) from why it exists (its selected-effect function) is a very important distinction in biology (Huneman 2013; Brunet and Doolittle 2014). Ignoring this distinction, and assuming that all genomic sites that exhibit a certain biochemical activity are functional, as was done by ENCODE Project Consortium (2012), is essentially equivalent to claiming that following a collision between a car and a pedestrian, a car’s hood would be ascribed the “function” of harming the pedestrian while the pedestrian would have the “function” of denting the car’s hood (Hurst 2013).

The ENCODE debate (Eddy 2012; Graur et al. 2013; Niu and Jiang 2013; Doolittle 2013; Palazzo and Gregory 2014) exposed the need for an evolutionary classification of genomic elements according to their selected effect function. Such a classification is also needed to dispose of the widespread misconception according to which evolutionary processes can ever produce a genome that is wholly functional. Actually, evolution can only produce such a genome if and only if 1) the effective population size is enormous—infinite to be precise, 2) the deleterious effects of increasing genome size by even a single nucleotide are considerable, and 3) the generation time is very short. Not even in the commonest of bacterial species on Earth are these conditions met. In species with small effective population sizes and long generation time, such as humans and perennial plants, a genome that is 100% functional is contrary to reason.

## The Classification

Our classification scheme starts with the premise that all genomes are the products of natural evolutionary processes, rather than intelligent design and, hence, contain both functional and nonfunctional parts. “Function” in the context of this article is understood as selected-effect function (Millikan 1989; Neander 1991, 2002; Graur et al. 2013). That is, a sequence is functional if it is maintained in the genome by natural selection because of its function. Furthermore, function is always defined in the present tense. In the absence of prophetic powers, one cannot use the potential for creating a new function as the basis for claiming that a certain genomic element is functional. For example, the fact that a handful of *Alu* elements have become functional cannot be taken as support for the hypothesis that all *Alu* elements are functional. The Aristotelian distinction between potentiality and actuality is crucial.

**Fig. 1.—:**
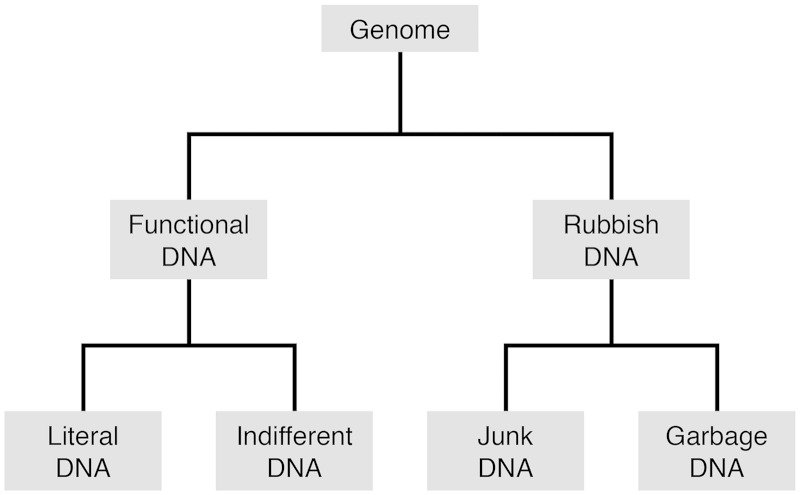
An evolutionary classification of genomic elements according to their selected-effect function.

We first divide the genome into functional DNA and rubbish DNA (fig. 1). “Functional DNA” refers to any segment in the genome whose selected-effect function is that for which it was selected and/or by which it is maintained. Most functional sequences in the genome are maintained by purifying selection. Less frequently, functional sequences exhibit telltale signs of either positive or balancing selection. There are many methods for identifying functional genomic segments under various selective regimes (e.g., Nielsen 2005; Vitti et al. 2013). “Low-level noncoding RNA transcription” (e.g., Kellis et al. 2014), for example, is not sufficient to assign functionality.

Functional DNA is further divided into “literal DNA” and “indifferent DNA.” In “literal DNA,” the order of nucleotides is under selection. Strictly, a DNA element of length *n* is defined as literal DNA if its function can be performed by a very small subset of the 4*^n^* possible sequences. For example, there are three possible sequences of length 3 that can encode isoleucine according to the standard genetic code, as opposed to the much larger number (64) of possible three-nucleotide sequences. Functional protein-coding genes, RNA-specifying genes, and untranscribed control elements are included within this category.

“Indifferent DNA” includes genomic segments that are functional and needed, but whose sequences are of little consequence. In other words, indifferent DNA refers to sequences whose main function is being there, but whose exact sequence is not important. They serve such functions as spacers, fillers, and protectors against frameshifts. The third codon position in 4-fold degenerate codons may be regarded as a simple example of indifferent DNA; the nucleotide that resides at this position is unimportant, but the position itself needs to be occupied. Some indifferent DNA may also serve nucleotypic functions, such as determining nucleus size (Cavalier-Smith 1978). Thus, indifferent DNA should show no evidence of selection for or against point mutations, but deletions and insertions should be under selection. For example, Nóbrega et al. (2004) deleted 2,356 kb from the mouse genome, yet mice homozygous for the deletions were indistinguishable from wild-type littermates with regard to morphology, reproductive fitness, growth, longevity, and a variety of parameters assaying general homeostasis. Thus, these sequences should be considered junk DNA rather than indifferent DNA.

“Rubbish DNA” (Brenner 1998) refers to genomic segments which have no selected-effect function. Rubbish DNA can be further subdivided into junk DNA and garbage DNA. The term “junk DNA” was current in the 1960s (e.g., Ehret and de Haller 1963); its meaning was formalized by Ohno (1972). Ohno’s definition of “junk DNA” refers to a genomic segment on which selection does not operate. Thus, junk DNA has no immediate use, although in the future it might acquire a useful function, albeit rarely. This sense of the word is very similar to the colloquial meaning of “junk,” such as when a person mentions a “garage full of junk,” in which the implication is that the space is full of useless objects, but that in the future some of them may be useful. Of course, as in the case of the garage full of junk, the majority of junk DNA will never acquire a function. Junk DNA and the junk in one’s garage are also similar in that “they may be kept for years and years and, then, thrown out a day before becoming useful” (Wool D, personal communication).

Because of linguistic prudery and the fact that “junk” is used euphemistically in off-color contexts, some biologists find the term “junk DNA” “derogatory” and “disrespectful” (Brosius and Gould 1992). An additional opposition to the term “junk DNA” stems from false teleological reasoning. Many researchers (e.g., Makalowski 2003; Wen et al. 2012) use the term “junk DNA” to denote a piece of DNA that can never, under any evolutionary circumstance, be selected for or against. As every piece of DNA may become functional and either become advantageous or deleterious by gain-of-function mutations, this type of reasoning is false. A piece of junk DNA may indeed be coopted into function, but that does not mean that it will be, let alone that it currently has a function.

“Garbage DNA” refers to sequences that exist in the genome despite being actively selected against. The reason that detrimental sequences are observable is that selection is neither omnipotent nor efficient. At any slice of evolutionary time, segments of garbage DNA (on their way to becoming extinct) may be found in the genome. Garbage DNA is expected to have a high turnover rate in evolution, but its disappearance from the genome is not instantaneous.

The distinction between junk DNA and garbage DNA was suggested by Brenner (1998):

“Some years ago I noticed that there are two kinds of rubbish in the world and that most languages have different words to distinguish them. There is the rubbish we keep, which is junk, and the rubbish we throw away, which is garbage. The excess DNA in our genomes is junk, and it is there because it is harmless, as well as being useless, and because the molecular processes generating extra DNA outpace those getting rid of it. Were the extra DNA to become disadvantageous, it would become subject to selection, just as junk that takes up too much space, or is beginning to smell, is instantly converted to garbage by one's wife, that excellent Darwinian instrument.”

Each of the four functional categories described above can be 1) transcribed and translated, 2) transcribed but not translated, or 3) not transcribed. Hence, we may encounter, for instance, junk DNA, junk RNA, and junk proteins.

It is beyond the scope of this article to discuss the relative amounts of literal, indifferent, junk, and garbage DNA in the genome. Theoretical considerations, however, lead us to believe that large genomes belonging to species with small effective population sizes and long generation times should contain considerable amounts of junk DNA and possibly quite a lot of garbage DNA too. Junk DNA is expected to persist in the genome for very long periods of evolutionary time; garbage DNA should be a more transient phenomenon.

## Changes in Functional Affiliation

**Fig. 2.—:**
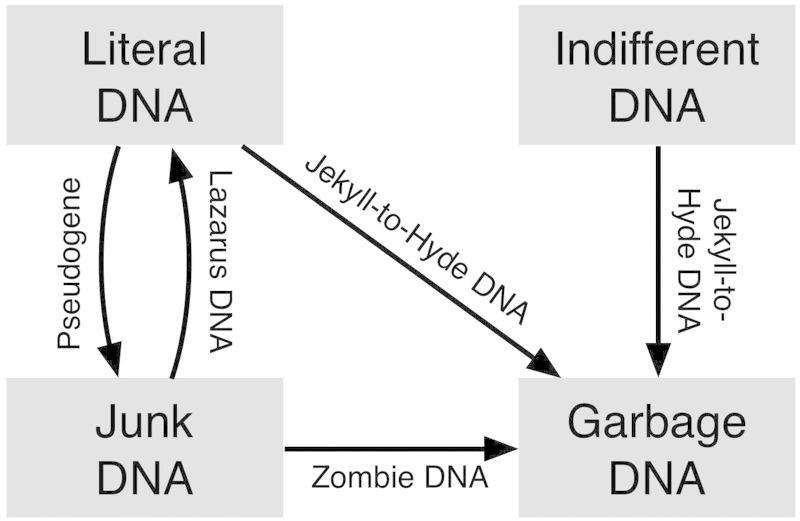
A suggested nomenclature for possible changes in the functional affiliation of genomic elements.

The affiliation of a DNA segment to a particular functional category may change during evolution. Because there are four functional categories, there may be 12 possible such changes (fig. 2). Several such changes are known to occur quite frequently. For example, junk DNA may become garbage DNA if the effective population size increases; the opposite will occur if the effective population size decreases (Ohta 1973). Many of the 12 possible changes have been documented in the literature. Here, we suggest a nomenclature for five such changes. Pseudogenes, for instance, represent a change in functional status from literal DNA to junk DNA, whereas some diseases are caused by either a change from functional DNA to garbage DNA (e.g., Chen et al. 2003) or from junk DNA to garbage DNA (Cho and Brant 2011). Rubbish DNA mutating to functional DNA may be referred to as “Lazarus DNA,” so named after the second most famous resurrected corpse in literature, Lazarus of Bethany (John 11:38–44; 12:1; 12:9; 12:17). Similarly, functional DNA may mutate to garbage DNA, in which case we suggest the term “Hyde DNA” based on the fictional transformation of a benevolent entity into a malicious one (Stevenson 1886). Alternatively, junk DNA may become garbage DNA, for which the term “zombie DNA” has been suggested (Kolata 2010).
